# Evaluation of the Potential Risk of Mortality from SARS-CoV-2 Infection in Hospitalized Patients According to the Charlson Comorbidity Index

**DOI:** 10.3390/healthcare10020362

**Published:** 2022-02-12

**Authors:** Jose Roberto Gutierrez-Camacho, Lorena Avila-Carrasco, Alberto Murillo-Ruíz-Esparza, Idalia Garza-Veloz, Roxana Araujo-Espino, Maria Calixta Martinez-Vazquez, Perla M. Trejo-Ortiz, Iram Pablo Rodriguez-Sanchez, Iván Delgado-Enciso, Maria E. Castañeda-López, Araceli Gamón-Madrid, Margarita L. Martinez-Fierro

**Affiliations:** 1 Molecular Medicine Laboratory, Unidad Académica de Medicina Humana y Ciencias de la Salud, Universidad Autónoma de Zacatecas, Zacatecas 98160, Mexico; robgtzc1987@gmail.com (J.R.G.-C.); doctoralac@gmail.com (L.A.-C.); idaliagv@uaz.edu.mx (I.G.-V.); calibgst@gmail.com (M.C.M.-V.); maru.casta77@gmail.com (M.E.C.-L.); araceli.gamon@uaz.edu.mx (A.G.-M.); 2 Instituto Mexicano del Seguro Social (IMSS), Hospital General de Zona No. 2, Fresnillo 99000, Mexico; albertomre05@hotmail.com; 3 Unidad Académica de Enfermería, Universidad Autónoma de Zacatecas, Zacatecas 98160, Mexico; roxana.araujo@uaz.edu.mx (R.A.-E.); perlu11@yahoo.com.mx (P.M.T.-O.); 4 Laboratorio de Fisiología Molecular y Estructural, Facultad de Ciencias Biológicas, Universidad Autónoma de Nuevo León, Monterrey 66450, Mexico; iramrodriguez@gmail.com; 5 Facultad de Medicina, Universidad de Colima, Colima 28040, Mexico; ivan-delgado-enciso@ucol.mx

**Keywords:** COVID-19, SARS-CoV-2, obesity, charlson index, comorbidity

## Abstract

**Background:** The pandemic of COVID-19 has represented a major threat to global public health in the last century and therefore to identify predictors of mortality among COVID-19 hospitalized patients is widely justified. The aim of this study was to evaluate the possible usefulness of Charlson Comorbidity Index (CCI) as mortality predictor in patients hospitalized because COVID-19. **Methods:** This study was carried out in Zacatecas, Mexico, and it included 705 hospitalized patients with suspected of SARS-CoV-2 infection. Clinical data were collected, and the CCI score was calculated online using the calculator from the Sociedad Andaluza de Medicina Intensiva y Unidades Coronarias; the result was evaluated as mortality predictor among the patients with COVID-19. **Results:** 377 patients were positive for SARS-COV-2. Obesity increased the risk of intubation among the study population (odds ratio (OR) = 2.59; 95 CI: 1.36–4.92; *p* = 0.003). The CCI values were higher in patients who died because of COVID-19 complications than those observed in patients who survived (*p* < 0.001). Considering a CCI cutoff > 31.69, the area under the ROC curve was 0.75, with a sensitivity and a specificity of 63.6% and 87.7%, respectively. Having a CCI value > 31.69 increased the odds of death by 12.5 times among the study population (95% CI: 7.3–21.4; *p* < 0.001). **Conclusions:** The CCI is a suitable tool for the prediction of mortality in patients hospitalized for COVID-19. The presence of comorbidities in hospitalized patients with COVID-19 reflected as CCI > 31.69 increased the risk of death among the study population, so it is important to take precautionary measures in patients due to their condition and their increased vulnerability to SARS-CoV-2 infection.

## 1. Introduction

The pandemic occasioned by the novel coronavirus 2019 (SARS-CoV-2) has represented the major threat to global public health in the last century [1,2,3]. The coronavirus 2019 disease (COVID-19) manifests itself mainly as fever, fatigue, cough [4,5] and, less frequently, the presence of upper respiratory tract symptoms, which may be due to the fact that the virus infects cells through angiotensin converting enzyme 2 (ACE-2), which is expressed mainly in cells of the lower respiratory tract [6,7]. Approximately half of the patients also develop dyspnea after one week [4], and in severe and critical cases, it progressed rapidly (average of 9 days) to acute respiratory distress syndrome (ARDS) with mild symptoms in the early stage [8]. The clinical presentation of COVID-19 is variable. In a general manner, older men (>60 years old) with comorbidities are more likely to develop a severe form of COVID-19, and they have a higher risk of dying from disease [9,10,11], whereas most young people and children present only the mild forms of disease, or they are asymptomatic [12]. 

The hypertension has been frequently reported as the main pre-existing comorbidity in COVID-19 patients [13,14] whereas that cardiovascular disease is also a common finding among COVID-19 patients [15]. According to a meta-analysis and systematic review on the risk of death in individuals with COVID-19 infection and hypertension, these patients had a significantly higher mortality risk compared to that observed in normotensive patients [14,15]. As an example, a study conducted at Wuhan Pulmonary Hospital and Jinyintan Centre Hospital revealed a mortality associated to patients with hypertension and COVID-19 of 28.27% [16].

Diabetes mellitus is the third most frequent underlying comorbidity in COVID-19 patients [17], and obesity, and obesity-related conditions, worsen the effect of SARS-CoV-2 infection [18,19] increasing the risk of COVID-19 complications [18,19,20,21]. The COVID-19 case-fatality rate was 10.5% in patients with cardiovascular disease, 6.0% in those with hypertension, and 7.3% in patients with diabetes mellitus [22]. Patients with diabetes mellitus who had other comorbidities showed higher case-fatality rates due to hyperglycemia [23]. 

Whereas the prevalence of chronic kidney disease (CKD) among COVID-19 patients is low, those with pre-existing CKD have been associated with severe COVID-19 and increased mortality [24]. Kidney injury has been found in more than 20% of critically ill patients by COVID-19, and these data have been supported by countries such as Italy [25], China [23], and the United States [26]. This is thought to be because the kidney has an increased vulnerability to SARS-CoV-2 infection due to the increased expression of ACE2 [27]. 

Mechanisms that may aggravate health conditions of patients with comorbidities infected with SARS-CoV-2 include chronical physiological changes related with chronical disease such as altered metabolism and/or immunity, which may lead to thrombosis and cytokine storm [28]. The host immune response to SARS-CoV-2 infection has an important role in the outcome of COVID-19 [29]. At the molecular level, SARS-CoV-2 infection leads to an inflammation state mainly manifested by an increase in interleukin (IL)-2, IL-7, interferon gamma (INFγ), and tumor necrosis factor alpha (TNFα) [30] and suppression of NF-kB (nuclear factor kappa B) activity, resulting in decreased expression of COX-2 (cyclooxygenase-2) [31]. IL-6 was found to be associated with highly pathogenic SARS-CoV-2 infection, due to enhanced virus replication mainly in the lower respiratory tract [32]. In patients with comorbidities including diabetes mellitus, the immune response may be not established correctly, and it may have an impact on the progression of COVID-19 [33]. In addition, other factors including components of the renin angiotensin aldosterone system (RAAS) may also be involved in the severity of COVID-19. For example, in normal conditions, the effects of ACE activation include vasoconstriction, oxidative stress, inflammation, and apoptosis, whereas the downstream effects of ACE2 activation include vasodilation, angiogenesis, anti-inflammatory, antioxidant, and anti-apoptotic effects. Consequently, considering that adipose tissue also expresses most of the components of the RAAS, such as angiotensinogen (AGT), angiotensin converting enzymes (ACE and ACE2), and their receptors, an imbalance or alteration in the production of the aforementioned factors as consequence of obesity and/or other comorbidities may aggravate or jeopardize the health condition of the patient infected with SARS-CoV-2 [34]. Patients with obesity are very prone to decreased airway flow due to limited truncal expansion, which hinders airflow and causes poor breathing; oxygen consumption and respiratory potential can be severely impaired and predispose to infections and the need for oxygen support, and there is a serious challenge for intubation [35]. Indicators to evaluate the progression and/or severity of COVID-19 include radiographic findings, indicators of organ dysfunction, and laboratory markers; however, the severity of the COVID-19 course is underscored by the fact that SARS-CoV-2 has tropism in various tissues, including the respiratory tract, kidney, brain, heart, endothelium, and liver [36].

Several studies have identified features for stratifying the risk of death of patients in the general population and/or with several conditions, but they are generally not used in clinical practice due to their complexity or lack of external validity [37]. The Charlson Comorbidity Index (CCI) is a simple standardized score used to predict mortality with respect to the weight of comorbidities in various pathologies [38,39]. The CCI score was developed in 1987 and has since been used in several studies to determine the impact of comorbidities on mortality prediction [40]. CCI is calculated from data collected from patient case studies, and the necessary information is obtained from the hospitals’ electronic database. Trained investigators must review and verify the electronic data collection forms for its establishment [41]. Considering that the CCI integrates features related with risk of death and that, at the present date, there are no data of predictors for COVID-19-related mortality, in this study, we aimed to evaluate the possible usefulness of CCI as a mortality predictor in patients hospitalized because of COVID-19. The importance of analyzing the comorbidities and the age of the hospitalized patients with COVID-19 through CCI to evaluate a potential mortality risk could guide medical staff to establish timely preventive and therapeutic strategies allowing a better prognosis of the patient.

## 2. Materials and Methods

### 2.1. Study Population

In this hospital-based retrospective cohort study, patients suspected of being infected with SARS-CoV-2 admitted to the Hospital General de Zacatecas “Luz González Cosío” (Zacatecas, Mexico) from March to October 2020 were enrolled. After hospital admission, each patient experienced nasopharyngeal exudate to confirm or rule out the presence of SARS-CoV-2 infection. Patients were selected only if they had criteria hospitalization and also signed the informed consent form. SARS-CoV-2 detection was done by real-time reverse transcriptase polymerase chain reaction (RT-PCR) assays as described previously [42]. 

The inclusion criteria for the study included patients over 18 years of age, with a positive RT-PCR test for SARS-CoV-2 and who were hospitalized in the General Hospital of Zacatecas and who were residents of the State of Zacatecas. Patients who were hospitalized for presenting symptoms related to COVID-19 but whose final diagnosis was another infectious disease were excluded. A trained team of health personnel reviewed and collected demographic, epidemiologic, medical history, treatment, clinical data, and the results of laboratory testing for SARS-CoV-2 for all patients since admission. Patients were considered with obesity when they had a body mass index (BMI) of ≥30 kg/m^2^; this was calculated by dividing the weight (kg) by the height squared (m²) [43]. 

### 2.2. Sample Size Calculation 

The size of sample calculated for the study was 385, and it was obtained online (https://www.questionpro.com/sample-size-calculator/ (accessed on 10 October 2020)), using the formula proposed by Murray and Larry (2005) [44,45] with a confidence level of 95%, *p* = 0.5, a margin of error of ±4%, and the population of the State of Zacatecas, which is 1,622,138 inhabitants.

### 2.3. Charlson Comorbidity Index (CCI)

The CCI is a method of predicted likelihood of death by classifying and/or weighting some comorbidities and has been widely used in health-related research to measure the burden of disease and case mix [46]. It is a validated, simple, and easily applicable method for estimating the risk of death from comorbid disease and has been used as a predictor of prognosis and long-term survival; it is therefore of utmost importance to have a thorough evaluation of comorbidities to establish risk stratification of patients with COVID-19 when they are admitted to the hospital [39]. 

The use of the CCI in the dialysis population has confirmed that high scores predict poor survival, poor health status, and an increase in the number of hospitalized patients; some reports have shown that the CCI had an index of concordance of 74% and is a suitable tool for the measurement of comorbidity in renal transplant recipients [47]. Researchers have evaluated CCI to predict short- and long-term mortality among those in emergency department patients with suspected infection or in nonsurgical emergency department patients [48,49]; likewise, it has been shown that CCI independently predicted mortality in acutely ill hospitalized patients [50]

Comorbidities were defined as the presence of one or more additional conditions existing simultaneously, independently or not, with a disease considered primary. The patient’s age and the prevalence of comorbid conditions are assigned in this index. The index score is obtained with the CCI calculator [51]. https://www.samiuc.es/indice-de-comorbilidad-de-charlson-cci/ (accessed on 11 November 2021).

### 2.4. Data Analysis

Continuous variables were expressed as mean values ± standard deviation. For the inferential statistics, a normal data distribution was first determined using the Kolmogorov–Smirnov test, and the equality of variances was confirmed using the Levine’s test. The comparison of two data series was carried out using an unpaired t-test for normally distributed data and the Mann–Whitney Rank Sum test to analyze non-normally distributed data. Categorical variables were expressed as frequency (percentage) and compared using the chi-square test. The odds ratio (OR) was calculated for each variable separately. Univariate statistical analysis was performed with the SPSS software package (version 13.0, SPSS Inc., Chicago, IL, USA). In a second stage and to evaluate the usefulness of the CCI to predict mortality among hospitalized patients with COVID-19, a Receiver Operating Characteristic curve (ROC) analysis was performed and after OR calculation was also considered to evaluate differences between groups categorized according CCI cutoff values. Both ROC and OR-derived analyses were carried out using Sigma Plot v.11. During all the statistical tests, significant values were considered those with *p* < 0.05. 

## 3. Results

In this study, 705 patients suspected of being carriers of SARS-CoV-2, aged between 18 and 93 years, were enrolled, of which 362 were women. RT-PCR was performed on these patients for virus detection. A total of 377 subjects were SARS-CoV-2 positive. Considering that CCI does not include obesity in their calculations, the study population was stratified as patients with COVID-19 with and without obesity (Table 1). In the study population, obesity was present in 157 (41.6%) patients. Table 1 shows symptoms, signs, and risk factors related to COVID-19 of the population substratified into groups of COVID-19 patients with obesity and COVID-19 patients without obesity. There were differences in the proportions of comorbidities such as T2DM or HAS between hospitalized patients with COVID-19 with and without obesity (*p* ≤ 0.05). Polypnea, thoracic pain, and dyspnea were the symptoms with differences in the proportions between study groups being more frequent in the group of COVID-19 patients with obesity (*p* < 0.05).

A total of 132 (35%) patients in the study died because of COVID-19 complications, and 55 (41.6%) of them had obesity. There were no differences in the proportions of patients who died because pf COVID-19 complications between groups of patients with and without obesity (*p* = 0.708). Regarding mechanical ventilation, considering the 377 patients with COVID-19, 45 (11.9) were intubated for mechanical ventilation, of which 28 (17.8%) had obesity. Obesity increased by 2.59 times the odds of intubation among the study population (95% CI: 1.364–4.924; *p* = 0.003). 

**Figure 1:**
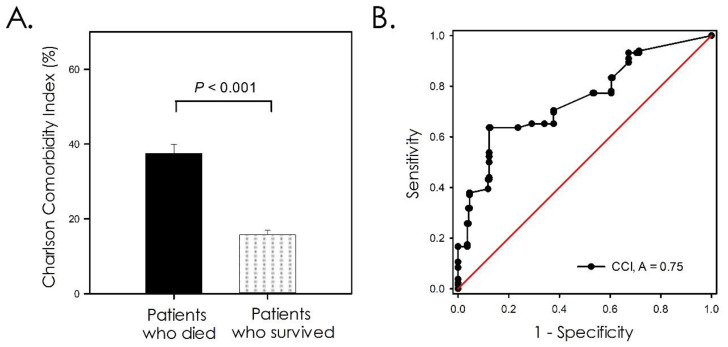
Charlson comorbidity index analysis. (**A**) Comparison between Charlson comorbidity index values between patients who died because of COVID-19 complications and of those who survived. (**B**) Value of Charlson comorbidity index as predictor of mortality between patients hospitalized because COVID-19. CCI: Charlson comorbidity index. A: Area under ROC curve.

To evaluate the potential association of CCI values and the mortality of hospitalized patients with COVID-19, the participants in the study were substratified as patients who died because of COVID-19 complications and those who survived. The result of this analysis is shown in Figure 1A. The CCI values were higher for patients who died because of COVID-19 complications (37.5 ± 28.2) than for those observed in patients who survived (15.7 ± 18.2), this difference being statistically significant (*p* < 0.001). To evaluate the CCI ability to predict mortality between hospitalized patients with COVID-19, an area under the ROC curve analysis was carried out. The results are shown in Figure 1B. Considering the total of the studied population, the ROC area was 0.75 (95% CI: 0.69–0.80). Considering a CCI cutoff > 31.69, the sensitivity of the method was 63.6% (95% CI: 54.8–71.8), and the specificity value was 87.7% (95% CI: 82.65–91.75). Having a CCI value > 31.69 increased the odds of death by 12.5 times among the studied population (95% CI: 7.3–21.4; *p* < 0.001). 

Table 2 shows the 132 patients (67 men and 65 women) with positive results in the SARS-CoV-2 screening test and those who died and the 220 (105 men and 105 women) patients with COVID-19 and those who survived. In this table, patients were grouped by age groups with 10-year intervals (except the first group). The table shows the average percentage of the CCI for each group of patients, which is related with the presence of comorbidities. 

It is important to note that, at the moment, there is no validated software for the calculation of this index that includes the COVID-19 in its calculations. In a general manner, the average of CCI of hospitalized patients who died from COVID-19 was higher compared to that observed for patients who survived. Higher values for CCI were observed for all the age groups, with the exception for the age group of 81–90 years old (Table 2).

## 4. Discussion

In the last year, studies have suggested that patients with other comorbidities are at high risk of developing more severe or severe COVID-19 [4]. The aim of this study was to evaluate the possible usefulness of CCI as a mortality predictor in patients hospitalized because of COVID-19. It is believed that the critical form of the disease and comorbidities such as obesity, hypertension, diabetes, cardiovascular disease, or respiratory disease could also greatly affect the prognosis of COVID-19 [52]. In agreement with our results, these comorbidities were risk factors for severe disease progression and even death [53]. 

As observed in previous reports, in our study, hospitalized persons with COVID-19 and with obesity required invasive mechanical ventilation more frequently, compared to patients without obesity, regardless of sex, age, arterial hypertension, or T2DM [54]. In the same way, our data also showed that the proportion of hospitalized COVID-19 patients with obesity and hypertension was higher than that observed in patients with another comorbidity. Previous studies suggest that patients with diabetes mellitus have an increased risk of infections, because of multiple alterations in innate immunity. Apparently, humoral immunity is not affected, and people with diabetes mellitus have deficits in neutrophil function and alterations in the phenomena of chemotaxis, adhesion, and phagocytosis and also in the intracellular destruction of pathogenic microorganisms [55]. Patients with T2DM usually have an excess of adipose tissue, which, according to pathophysiological processes, can trigger an alteration of glycemic homeostasis, produce some important alterations such as chronic inflammatory state and chronic hyperglycemia, which cause a negative effect on patients with respect to immunity and make them more susceptible to infections—in this case, to the infection caused by SARS-CoV-2 [56,57]. 

**Figure 2:**
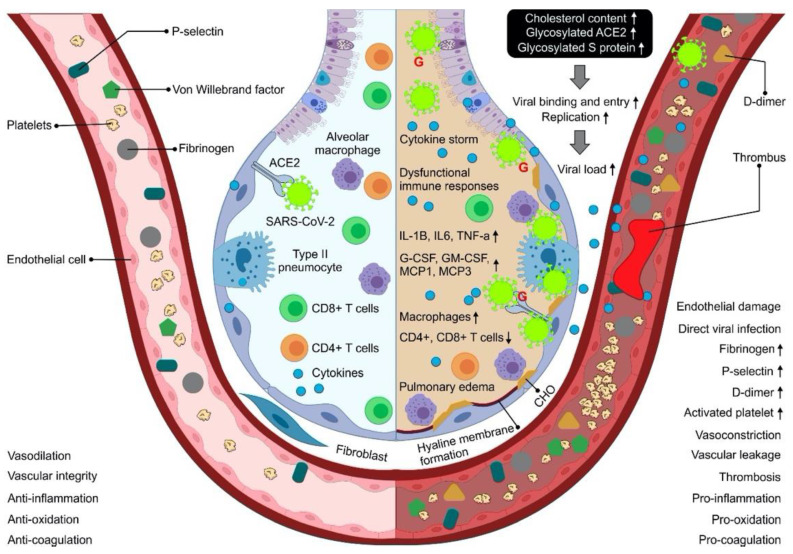
Pathophysiological mechanisms related with severe COVID-19 in patients with and without comorbidities. Following SARS-CoV-2 infection, the acquired and innate immune responses to that of people without comorbidities are effectively activated to eliminate the pathogen and infected cells with minimal inflammation and lung damage. The integrity of their vascular endothelial cells is well maintained, and the vessels possess normal functions of contraction and dilation, anti-inflammation, anticoagulation, and antioxidant capacity. However, patients with comorbidities such as hypertension, cardiovascular disease, obesity, and/or diabetes have an increased viral load when exposed to an amount of virus equivalent to those people without comorbidities. Impaired antiviral immunity, leaky SARS-CoV-2 infections, and excessive macrophage infiltration together contribute to uncontrolled cytokine storm, promoting the development of immunopathology such as pulmonary edema and hyaline membrane. The predisposition to a prothrombotic state in COVID-19 is associated with chronical conditions, driven largely by endothelial damage, platelet hyperactivation, hypercoagulability, hyperinflammation, and impaired fibrinolysis and poor outcomes [9,30,31,32,57]. CHO: cholesterol; G: glycosylated; SARS-CoV-2: severe acute respiratory syndrome coronavirus 2; COVID-19: coronavirus disease 2019.

The molecular mechanisms associated with SARS-CoV-2 infection involves an increase in inflammation, leading to an increase in cytokines (cytokine storm) in patients with COVID-19 [30], mainly manifested by an increase in interleukin IL-2, IL-7, INFγ, and TNFα, leading to suppression of NF-kB activity, resulting in decreased expression of COX-2 [31]. Among all interleukins, IL-6 was found to be associated with highly pathogenic SARS-CoV-2 infection, due to enhanced virus replication mainly in the lower respiratory tract [32,57] (Figure 2).

In the course of this pandemic, severity and mortality caused by COVID-19 are generally predicted by sex, age, and the presence of comorbidities, such as cardiovascular disease, diabetes mellitus, and respiratory diseases [58,59,60,61,62]. To predict the risk of mortality in hospitals, the CCI was developed, and it is based on the score of a series of comorbidities, which have a weighted assigned number from 1 to 6, which is directly related to the severity of morbidity; CCI also predicts the survival rate of patients in the next 10 years [63]. This well-validated index is easy and simple to apply to assess patient survival and prognosis. In addition to the benefits of this index mentioned above, we believe that the most important is the optimization of time by performing it at the time of hospitalization, since it covers an acceptable number of comorbidities. The disadvantages of the other prognostic evaluation calculators are, for example, that the LEE Prognostic Index currently has only five variables that we could relate to COVID-19 [64]. The APACHE-II Index is useful for classifying patients by recording 12 physiological parameters and is therefore not useful in this study [65,66]. The SAPS Index also evaluates the deviation of some physiological values to determine with the results if the severity of the disease increases or not, so it is not useful for us either [67]. There is also the Mortality Prediction Models (MPM-II) that predicts mortality or survival of patients at the time of admission to the Intensive Care Unit; its disadvantage is the exclusion of several morbidities and the use of variables that would not be useful to compare this study [68,69]. Our results showed that the CCI is a valid and reliable tool for predicting general mortality among hospitalized patients with COVID-19 [70]. 

Age is another variable related with severity in COVID-19 and in the mortality observed in the previous pandemic related with SARS infection [71,72]. Older age has been considered as an independent predictor of mortality; age-dependent immune cells leading to a stronger inflammatory response have been suggested as a theory for increased mortality in the elderly [73]. In a systematic review, researchers showed that higher CCI was related with elevated mortality and disease severity in COVID-19 patients [39]. Multimorbidity captured by the CCI has been found to have a stronger relationship with mortality than almost all individual comorbid conditions and also has the advantage that it is calculated directly from diagnosis codes obtained from administrative medical data; it is widely used across numerous diseases, health care systems, and populations [74]. However, a CCI score greater than 0 was related with an elevated risk of severe COVID-19 and death when controlled for age and sex, extending previous findings of individual comorbidities as independent risk factors for poor COVID-19 outcomes [75]. Likewise, in another study, it was shown that age and comorbidities are the most significant predictors of death among COVID-19 patients. In agreement with those results, in our study, the presence of multiple comorbidities reflected as a CCI higher than 31.69 increased the odds of death by 12.5 times among the studied population. It has been found that the CCI score can exponentially predict all non-surviving patients and could represent a powerful screening for requesting hospital admission after COVID-19 diagnosis [76]. A high score could have a very important significance not only for predicting unfavorable outcomes in SARS-CoV-2 infected patients but also for better identifying individuals who could benefit from stricter isolation measures, quarantine, and earlier prevention strategies or, in case of infection, earlier treatment [76]. 

Hospital mortality due to COVID-19 is highly variable as various factors are involved [77] such as the way in which the case is defined, the number and type of comorbidities, the patient case mix, the geographic location, and the capacity of health systems around the world [78]. A variance in case fatality in elderly patients has been described around the world, where case fatality in this group of patients has been reported to range from 14% to 35% [77,79,80,81,82]. In our study in the age group of 81–90 years, there was no statistical significance in the comparison of CCI, probably due to the similarity in number and type of comorbidities coupled with obesity among patients who died and survived COVID-19.

Finally, it is important to highlight that the main limitations of this study are the lack of laboratory results of the patients, the outcome of the patients who requested voluntary discharge before overcoming the disease, and the non-inclusion of the severity of symptoms caused by COVID-19. These data would have possibly been useful for evaluating their weighting among the clinical variables and risk factors related with COVID-19. In the same way, today, vaccination campaigns around the world have been the first method to counteract the COVID-19 pandemic and all its consequences, including hospitalizations and deaths. One of the considerations to note is that the hospitalized patients included in our study had not received SARS-CoV-2 vaccine because it was not yet available at the time of data collection. The distribution and use of vaccines are of utmost importance to avoid the severity of symptoms as much as possible, and according with our results, to encourage booster vaccines, especially in the elderly and vulnerable groups, should be a highly recommended strategy worldwide [83].

## 5. Conclusions

Obesity increased the risk of intubation 2.59 times in the study population. The presence of comorbidities in hospitalized patients with COVID-19 reflected as CCI > 31.69 increased the risk of death by 12.5 times in the studied population, so it is important to take precautionary measures in patients due to their general health condition and their increased vulnerability against SARS-CoV-2 infection. The CCI is a suitable tool for the prediction of mortality in patients hospitalized for COVID-19. Understanding the importance of the presence of comorbidities and the risk of severe COVID-19 should be a public health priority since the prevalence of these conditions are high in the Mexico and in the world. 

## Figures and Tables

**Table 1:** Comparison of findings between patients with COVID-19 with and without obesity (*n* = 377).

Variable	Patients with COVID-19 and Obesity (*n* = 157)	Patients with COVID-19 without Obesity (*n* = 220)	Odds Ratio (95% CI)	*p*-Value
Risk factors				
Type 2 diabetes mellitus	49 (31.2)	49 (22.2)	1.583 (0.996–2.517)	**0.05**
COPD	10 (6.3)	16 (10.1)	0.867 (0.383–1.965)	0.733
Tobaccoism	12 (7.6)	28 (12.7)	0.567 (0.279–1.154)	0.114
Chronic renal disease	6 (3.8)	3(1.3)	2.874 (0.708–11.671)	0.123
Heart disease	3 (1.9)	10 (4.5)	0.409 (0.111–1.511)	0.167
HIV/AIDS	0 (0.0)	1(0.4)	N.A.	-
Asthma	4 (2.5)	4 (1.8)	1.412 (0.348–5.732)	0.628
Immunosuppression	10 (6.3)	12 (5.4)	1.179 (0.496–2.802)	0.709
Hypertension	72 (45.8)	71 (32.2)	1.778 (1.165–2.712)	**0.007**
Symptoms				
Anosmia	62 (39.4)	83 (37.7)	1.077 (0.708–1.640)	0.729
Dysgeusia	62 (39.4)	72 (32.7)	1.342 (0.876–2.055)	0.176
Cyanosis	42 (26.7)	50 (22.7)	1.242 (0.773–1.994)	0.370
Conjunctivitis	8 (5.0)	17 (8.5)	0.641 (0.270–1.525)	0.311
Abdominal pain	39 (24.8)	44 (20.0)	1.322 (0.810–2.158)	0.263
Vomiting	23 (14.6)	25 (11.3)	1.339 (0.729–2.458)	0.345
Polypnea	101 (64.3)	110 (50.0)	1.804 (1.185–2.745)	**0.006**
Fever	136 (86.6)	179 (81.3)	1.483 (0.838–2.626)	0.174
Myalgia	113 (71.9)	154 (70.0)	1.101 (0.700–1.730)	0.678
Arthralgia	102 (64.9)	136 (61.8)	1.145 (0.748–1.754)	0.532
Rhinorrhea	37 (23.5)	61 (27.7)	0.804 (0.501–1.289)	0.364
Attack to the general state	137 (87.2)	176 (80.0)	1.713 (0.965–3.040)	0.064
Headache	126 (80.2)	186 (84.5)	0.743 (0.434–1.271)	0.277
Calophries	83 (52.8)	110 (50.0)	1.122 (.744–1.690)	0.583
Diarrhea	40 (25.4)	53 (24.0)	1.077 (0.671–1.730)	0.758
Thoracic pain	103 (65.6)	120 (54.4)	1.590 (1.041–2.426)	**0.031**
Cough	140 (89.1)	189 (85.9)	1.351 (0.719–2.538)	0.349
Odynophagia	83 (52.8)	115 (52.2)	1.024 (.680–1.543)	0.909
Dyspnea	132 (84.0)	158 (71.8)	2.072 (1.233–3.480)	**0.005**
Irritability	11 (7.0)	13 (5.9)	1.200 (0.523–2.752)	0.667
Mechanical ventilation (Intubation)	28 (17.8)	17 (7.7)	2.592 (1.364–4.924)	**0.003**

Data are displayed as frequency and percentage. *p*-values < 0.05 are highlighted in bold. NA = Not applicable.

**Table 2:** Comparison of the Charlson comorbidity index by age group in hospitalized patients who did and did not die because of COVID-19 complications.

Age Group (Years)	Patients Who Died of COVID-19 (*n* = 132)	Charlson Comorbidity Index *	COVID-19 Survivors (*n* = 220)	Charlson Comorbidity Index *	*p*-Value
18–30	4	2.9 ± 0.65	10	1.7 ± 0.86	**0.013**
31–40	4	3.3 ± 1.40	35	1.75 ± 0.37	**0.008**
41–50	21	7.1 ± 3.46	43	4.8 ± 0.73	**0.003**
51–60	34	30.02 ± 7.55	53	10.11 ± 1.4	**0.005**
61–70	30	42.12 ± 5.58	48	25.4 ± 4.15	**0.007**
71–80	22	63.5 ± 11.37	17	29.4 ± 11.03	**0.001**
81–90	15	61.5 ± 14.30	11	62.22 ± 11.33	0.661
91–100	2	88.19 ± 55.46	3	78.54 ± 10.30	**0.020**

* Charlson comorbidity index data are represented as mean of percentage by age group ± standard deviation. *p*-value was obtained from the comparison of the average of CCI score of the patients who died because COVID-19 complications and COVID-19 survivors using *t*-test. *p*-values < 0.05 are highlighted in bold.

## Data Availability

Additional data that support the findings of this study are available from the corresponding author (M.L.M.-F.), upon reasonable request.
